# Miscibility Studies of Bismesogen CBnCB Forming Nematic Twist-Bend Phase with Cyanobiphenyls nCB

**DOI:** 10.3390/ma17174256

**Published:** 2024-08-28

**Authors:** Marzena Tykarska, Barbara Klucznik, Jerzy Dziaduszek, Stanisław Jóźwiak

**Affiliations:** 1 Institute of Chemistry, Military University of Technology, ul. gen. Sylwestra Kaliskiego 2, 00-908 Warsaw, Poland; barbara.klucznik@wat.edu.pl (B.K.);; 2 Institute of Materials Science and Engineering, Military University of Technology, ul. gen. Sylwestra Kaliskiego 2, 00-908 Warsaw, Poland; stanislaw.jozwiak@wat.edu.pl

**Keywords:** liquid crystals, phase diagrams, miscibility studies, nematic twist-bend phase

## Abstract

This work aims to determine how the nematic twist-bend phase (N_TB_) of bismesogens containing two rigid parts of cyanobiphenyls connected with a linking chain containing n = 7, 9, and 11 methylene groups behaves in mixtures with structurally similar cyanobiphenyls nCB, n = 4–12, 14. The whole phase diagrams are presented for the CB7CB-nCB system. For the other systems, CB9CB-nCB and CB11CB-nCB, only curves corresponding to N_TB_-N phase transition are presented. Based on the temperature-concentration range of the existence of N_TB_ phase, it was established that an increase in the alkyl chain length of CBnCB causes an increase in the stability of the N_TB_ phase. But surprisingly, an increase in the alkyl chain length of nCB compounds does not change the slope of the N_TB_-N equilibrium line on phase diagrams. It is slightly bigger when the nCB compound has the same length of alkyl chain as the length of the linking group of a bismesogen. XRD studies were carried out for two mixtures.

## 1. Introduction

Miscibility studies are often performed because they help in the identification of phases [1,2,3], allow us to establish the influence of the molecular structure on phase existence [4,5] and to check if compounds are good candidates for mixture components [6,7], because for application purpose mixtures are used. The results of miscibility studies of compounds are presented in phase diagrams, showing phase transition temperatures versus concentration. This is a tool that helps verify the influence of the composition of liquid crystal mixtures on phase situations. The phase may be supported, destabilized, or induced in mixtures [5,7,8]. Even compounds with non-compatible structures are mixed together to check the possibilities of phase formation [9,10,11,12].

**Figure 1:**
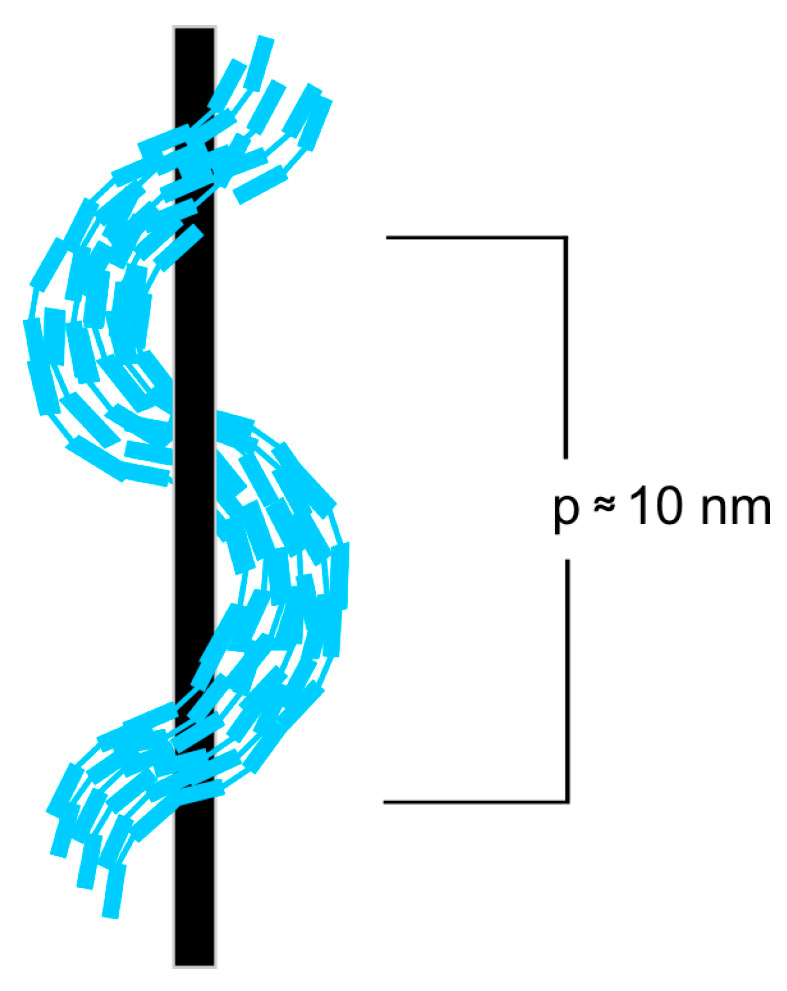
Structure of a twist-bend nematic N_TB_ phase; p–pitch of heliconical structure.

The liquid crystal nematic phase (N) is formed by elongated molecules with only an orientational order and translating along their long axes. When molecules contain a carbon atom with four different substituents, a chiral nematic phase is formed (N*) because molecules create a macroscopic helix. In 2001, Dozov predicted the possibility of forming a new nematic phase, namely the nematic twist-bend phase (N_TB_) [13]. Ten years later, the existence of this phase was experimentally confirmed [14]. In contradiction to the chiral nematic phases, in an N_TB_ phase, heliconical structure is formed by achiral molecules and their order is presented in Figure 1.

The first compound for which the N_TB_ phase was confirmed was 1,7-bis(4-cyanobiphenyl-4’-yl)heptane (CB7CB) [14]. This compound consists of two cyanobiphenyl groups linked by an alkyl chain. This compound is the most tested compound forming the N_TB_ phase, e.g., refs. [15,16,17,18,19,20]. An examination of CBnCB series of bismesogens shows that only compounds containing an odd number of methylene groups (n = 5, 7, 9, 11) in the linking alkyl chain form the N_TB_ phase [21]. More information about the N_TB_ phase can be found, e.g., in refs. [22,23,24,25,26].

Miscibility studies are performed to test the influence of the molecular structure of different compounds on maintaining the phases. In the literature, we may find examples of miscibility studies of CB7CB with different bismesogens (e.g., refs. [27,28,29]) and also with monomesogens which are chemically very similar, namely pentylcyanobiphenyl 5CB [30,31,32] and octylcyanobiphenyl 8CB [33,34]. On phase diagrams, the melting points are not presented so we cannot define the temperature range in which the N_TB_ phase is enantiotropic, as it is essential for the possibilities of making measurements. Recently, we reported the results of miscibility studies of the CB9CB compound with cyanobiphenyls nCB, n = 4–12, 14 [35]. The whole phase diagrams were presented there. The crystal phase is stabilized in mixtures more so than in theoretical predictions.

The aim of this work is to determine the influence of cyanobiphenyls nCB (n = 4–12, 14) on miscibility with CBnCB (n = 7, 9, 11) compounds. The whole phase diagrams are presented for the system CB7CB-nCB, but for CB9CB-nCB and CB11CB-nCB, only lines corresponding to the upper limit of existence of N_TB_ and SmA_d_ phases are compared. The presented results will enable us to test the influence of the alkyl chain length of tested compounds on the stability of the N_TB_, SmA_d,_ and crystal phases. This type of research fits into the topic of determining the influence of the structure of compounds on the formation of liquid crystalline phases.

## 2. Materials and Methods

**Figure 2:**
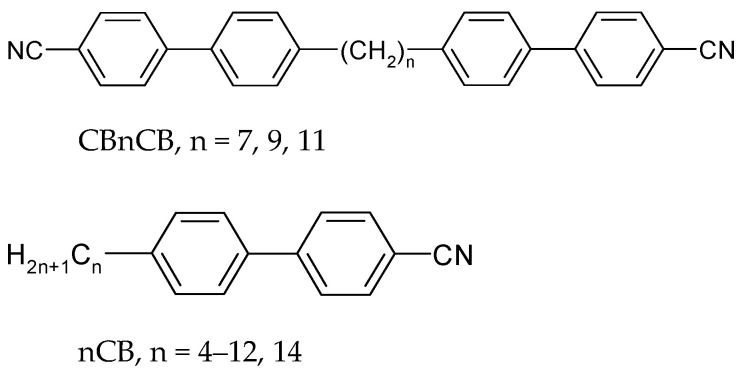
Chemical structure of tested compounds.

The chemical structures of examined compounds CBnCB and nCB are shown in Figure 2. Their phase transition temperatures are given in Table 1. The CBnCB compounds consist of two cyanobiphenyl groups linked with a chain of several methylene groups (n = 7, 9, 11). They form a twist-bend nematic phase N_TB_. The compounds that are mixed with CBnCB are cyanobiphenyls nCB, which have different alkyl chain lengths n = 4–12 and 14. Compound n = 4 forms a monotropic N phase, compounds n = 5–7 form an enantiotropic N phase, compounds n = 8 and 9 form SmA_d_ and N phases, and compounds n = 10–12, and 14 form only an SmA_d_ phase of a dimeric structure (SmA_d_ [36]).

The phase transition temperatures were determined from observations of texture changes with a polarizing optical microscope (POM) MP-349 (OPTA-TECH, Warsaw, Poland) equipped with heating stage THMSE6-200 (Linkam Scientific Instruments Ltd., Tadworth, UK) and temperature controller T95-STD (Linkam Scientific Instruments Ltd., Tadworth, UK). The measurements were performed in a heating cycle with a heating rate of 2 K/min near the phase transition region. The results are presented in the form of phase diagrams.

The phase transition enthalpies were measured using differential scanning microcalorimeter DSC 141 (Setaram, Caluire-et-Cuire, France) at both heating and cooling cycles with a scan rate of 2 K/min. This heating and scanning rate is optimal because one smaller than this is too small due to instruments’ limitations which cause transitions to be stretched in time, and a bigger one causes a shift in transition temperatures. The X-ray diffractograms were recorded on Ultima IV (Rigaku, Tokyo, Japan) using Co Kα_1_ radiation (λ = 1.789 Å) in the range of 2θ between 5° and 40°. The measurement was carried out in the reflection mode, using a parallel beam (Rigaku patent) with a scanning step of Δ2θ = 0.02° and a scanning speed of 1 °/min using a lamp voltage of 40 kV and a current of 40 mA. The tests were carried out at a constant, small beam incidence angle of 5°. The sample was placed in an open holder made of glass. The test temperature was 23 °C and was controlled using a thermohygrometer placed in the measuring chamber of the diffractometer.

## 3. Results and Discussion

### 3.1. Compound CB7CB

**Figure 3:**
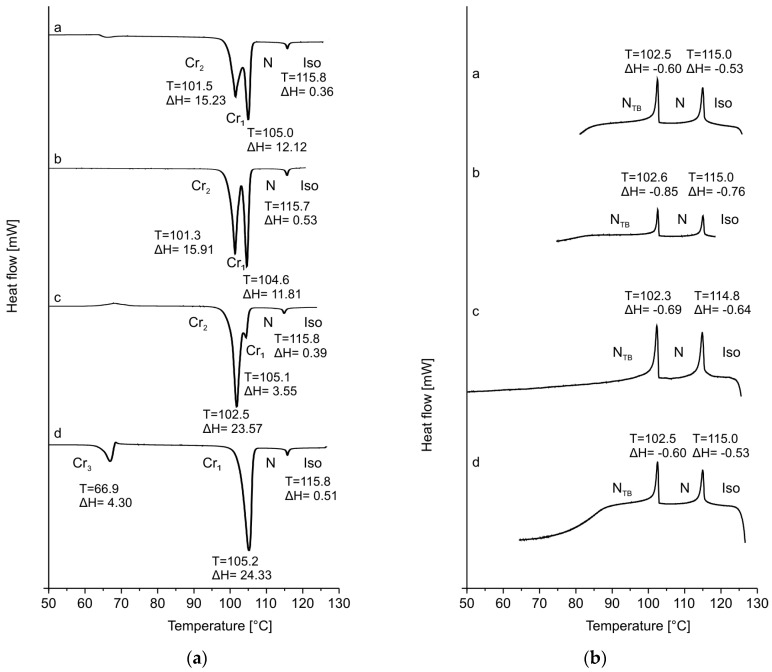
DSC thermograms of CB7CB compound: (**a**) under heating (a–c, the first heating, d, the second heating), (**b**) under cooling (a–c, the first cooling, d, the second cooling); scan rate 2 K/min. Presented T in [°C] and ΔH in [kJ/mol].

**Figure 4:**
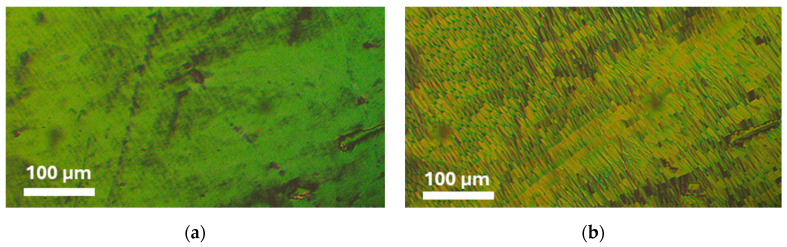
Micrographs of compound CB7CB: (**a**) at 108.8°C in nematic phase; (**b**) at 102.6 °C in twist-bend nematic phase, placed between untreated slides and crossed polarizers.

When the CB7CB compound was first reported in 1993, its phase transition temperatures were as follows: Cr 101 S 102 N 115 Iso [37]. The N_TB_ phase was incorrectly identified as a smectic phase there because the N_TB_ phase gives textures similar to those observed for the smectic phase. When the existence of the N_TB_ phase was experimentally confirmed in this compound in 2011, the transition temperatures were given as follows: Cr 102 N_TB_ 103 N 116 Iso [14]. In the case of our sample of the CB7CB compound, the phase transition temperatures are shown in Table 1 and Figure 3. The textures of the N and N_TB_ phases are given in Figure 4. We performed a few DSC measurements of the CB7CB compound with a heating/cooling rate of 2 K/min and found that this compound had three different crystal phases. They do not appear in every case. Figure 3a,b present the DSC thermograms obtained by heating and cooling. Curves a–c in Figure 3a obtained from the first heating of different samples show that two crystals Cr_2_ and Cr_1_ melt at 102.5 and 105.0 °C (peak top of the most separated peaks), respectively. The intensity of these peaks is different depending on the proportion of the existence of both phases in a sample. The d curve obtained from the second heating shows that the Cr_3_ phase melts at 66.9 °C and the Cr_1_ phase at 105.2 °C. The Scanning Tunneling Microscopy measurements show three different possible arrangements of bismesogenic molecules [44]. It is worth noticing that the sum of enthalpies of these different transitions is comparable (27.35, 27.72, 27.12, and 28.63 kJ/mol from curves a, b, c, and d, respectively). Thus, the disappearance of the crystal phase and formation of the liquid crystal phase require the same amount of energy regardless of the type of crystal phases formed. The N-Iso transition is at 115.8 °C, but in the cooling process, it is a little lower (115.0 °C). This is normal behavior because the transitions occurring during cooling are driven not only thermodynamically (as during heating) but also kinetically. The N_TB_-N transition is not visible in the DSC presented in Figure 3a, only in Figure 3b during cooling. However, as was mentioned, only transition temperatures measured during heating are characteristic parameters of materials. Such a thermogram, obtained for the CB7CB heated before crystallization appeared (presented in Figure 8a as 0 wt.% of 9CB), shows that the N_TB_-N transition is at 103.1 °C (the same as in ref [14]).

### 3.2. Miscibility Studies

#### 3.2.1. Influence of Alkyl Chain Length of nCB on Phase Situation

**Figure 5:**
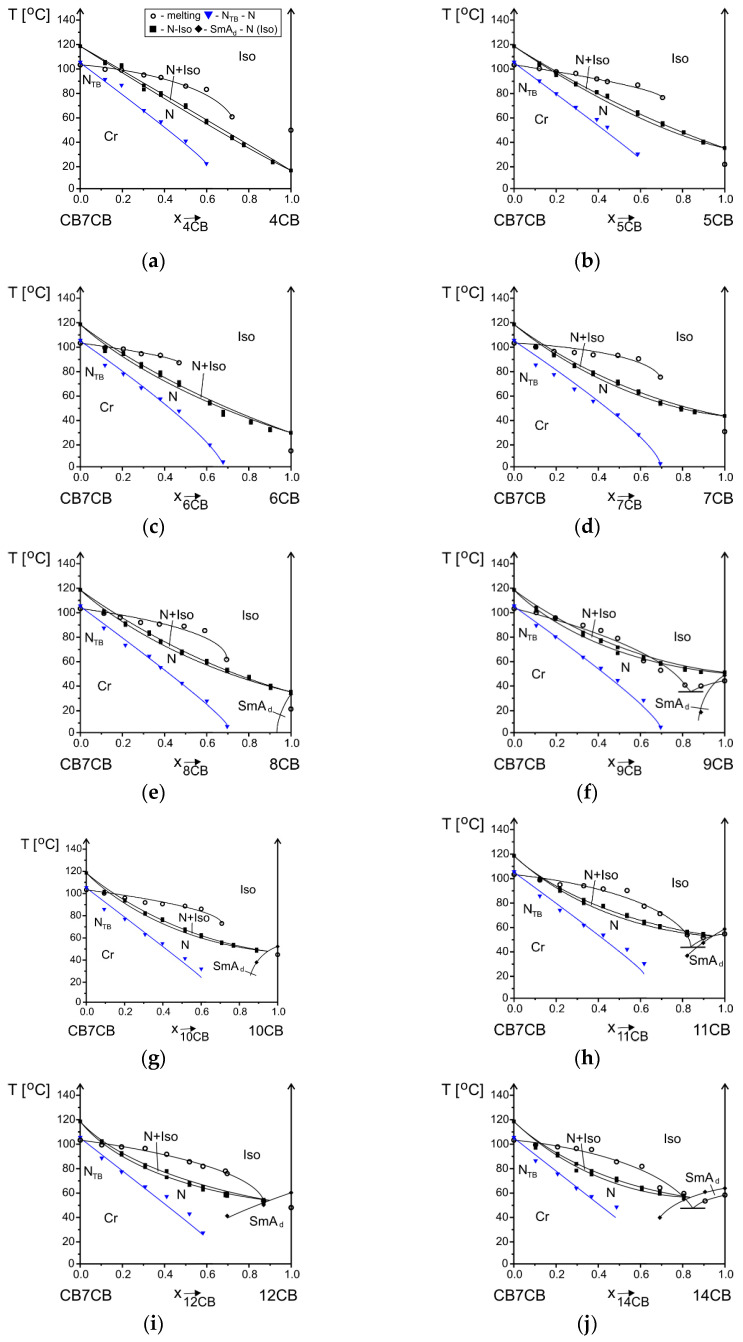
Phase diagrams presented for the CB7CB-nCB systems, n = 4 (**a**), 5 (**b**), 6 (**c**), 7 (**d**), 8 (**e**), 9 (**f**), 10 (**g**), 11 (**h**), 12 (**i**), 14 (**j**). Transition temperatures measured upon heating. Legend in (**a**) common for all graphs.

The CB7CB compound was mixed with nCB compounds (n = 4–12, 14) with a rigid core the same as one part of this bismesogen. The miscibility results are shown in phase diagrams, where phase transition temperatures are marked versus the concentration of the compounds (Figure 5). The concentration is given as a mole fraction; thanks to this, we can compare the results for different homologs. For example, a concentration of 0.5 mole fraction corresponds to a 1:1 ratio of both types of molecules. To achieve such acondition we have to prepare a mixture 40.2 wt.% of 9CB and only 34.1 wt.% of 4CB. All transitions were obtained in a heating cycle, even for the monotropic phase. After cooling, the samples and formation of the N_TB_ phase, before it was crystallized, it was heated.

The situation with **clearing transition** is different and depends on the kind of mesophases formed by cyanobiphenyls. When compounds nCB form a nematic phase, whether monotropic (n = 4) or enantiotropic (n = 5–9), it mixes additively with a nematic phase of CB7CB, and a biphase region (N + Iso) is observed within a temperature range with a maximum of 3 degrees. When compounds nCB form only the smectic A_d_ phase (n = 10–12, 14), the nematic and SmA_d_ phases undergo an eutectic transition. The temperature range of the biphase regions is too small to be well presented.

**Figure 6:**
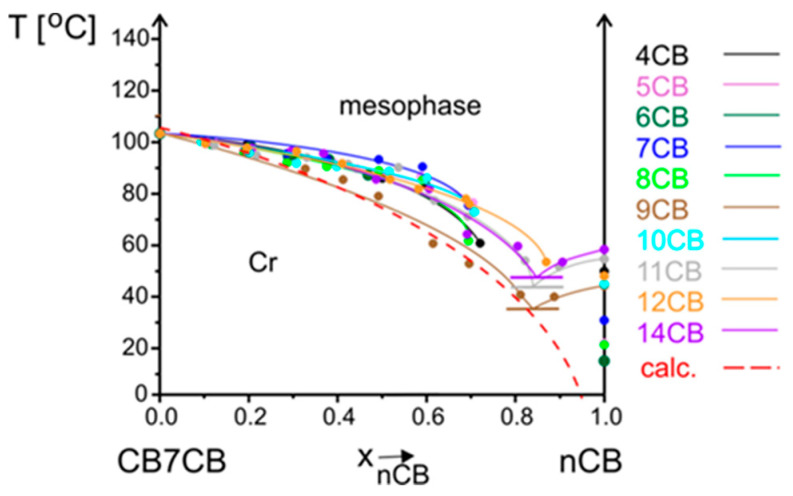
Melting temperatures for CB7CB-nCB system (n = 4–12, 14) versus concentration x (mole fraction). The results of the CSL calculations are marked with a dashed line.

The **crystal phase** melts according to a typical eutectic transition. The temperature of an eutectic mixture is marked with a short horizontal line. In all cases, an eutectic concentration is close to the nCB compound. For systems containing nCB n = 9, 11, 14, the crystallization was in the whole concentration range, but the other systems did not crystallize with a higher concentration of cyanobiphenyls. This is why the melting temperatures are not marked in a full concentration range. A comparison of melting temperature versus the concentration of compounds is presented in Figure 6. The calculated melting curve according to Le Chatelier, Schröder, and Van Laar (CSL) Equation (1), satisfying condition (2), is marked with a dashed line.

(1) $$\ln x_{k}=\frac{{∆H}_{m}^{k}}{R}(\frac{1}{T}-\frac{1}{T_{m}^{k}}),$$

(2) $$\sum_{k=1}^{n}x_{k}=1$$

**Figure 7:**
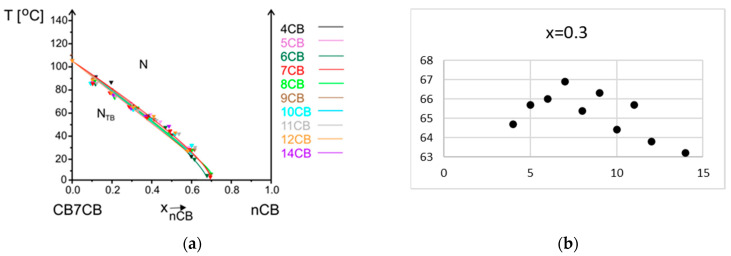
Phase transition N_TB_-N temperatures for CB7CB-nCB system (n = 4–12, 14) versus (**a**) concentration x (mole fraction), (**b**) alkyl chain length n for set concentration x = 0.3 mole fraction of nCB.

**Figure 8:**
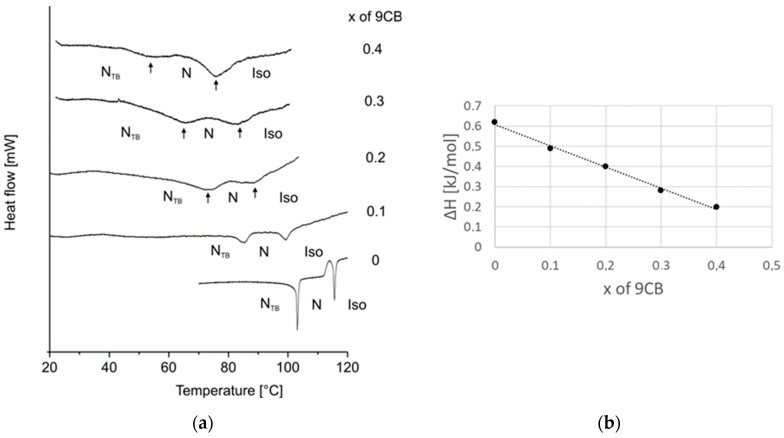
(**a**) DSC thermograms of CB7CB-9CB system; second heating started at temperatures before crystallization of first cooling was started. Concentration “0” corresponds to pure CB7CB; (**b**) changes in N_TB_-N transition enthalpies versus concentration in this system.

**Figure 9:**
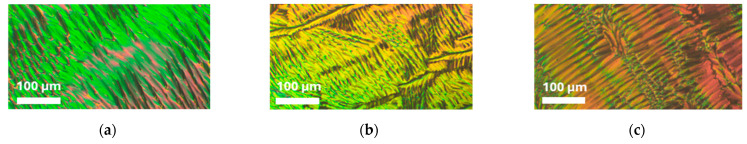
Micrographs of twist-bend nematic phase observed for mixtures CB7CB-9CB containing (**a**) 0.3 at 57.3 °C; (**b**) 0.4 at 49.8 °C; (**c**) 0.5 at 44.2 °C mole fractions of 9CB. Placed between untreated slides and crossed polarizers.

The measured melting temperature for most systems is observed to be higher than calculated. The crystal phase is more stable than the theory predicts; thus, intermolecular interactions between molecules in mixtures are stronger than in single compounds. The biggest difference is for the system with 7CB compound, which only slightly decreases the melting temperature, and up to 0.6 mole fraction, it is similar to the pure CB7CB compound. The **N_TB_ phase** is monotropic; thus, it can be reached during the cooling process from a nematic phase. However, the phase transition temperature measured upon cooling is not a characteristic parameter of a material. This is why, after cooling the sample at least 5 degrees below this transition and before crystallization started, the temperature was increased and N_TB_-N transition temperature was measured upon heating. The lines corresponding to the equilibrium between these two phases versus concentration are compared in one summary phase diagram presented in Figure 7a. The stability of the N_TB_ phase of the CB7CB compound decreases in mixtures upon an increase in the concentration of nCB compounds. These lines have the same slope. The same observation was made for systems where a longer bismesogen CB9CB was doped with cyanobiphenyls of different alkyl chain lengths [35]. The dependence of the temperature of maximum stability of the N_TB_ phase upon the alkyl chain length of nCB compounds, for concentration x = 0.3 mole fraction of nCB, is presented in Figure 7b. Even though the values are within 4 degrees, the odd–even effect in the results can be noticed. The maximum temperature of the N_TB_-N transition is for the system where the 7CB compound is added to CB7CB. For systems CB9CB-nCB, the maximum was observed for 9CB [35]. The mentioned observations show that the most stable N_TB_ phase is obtained when the alkyl chain length of cyanobiphenyl has the same length as the linking alkyl chain of a bismesogen. Molecules can fit better into the structure formed by a bismesogen. The maximum concentration of the existence of the N_TB_ phase is mostly up to a 0.6–0.7 mole fraction of nCB. The exact border concentrations of N_TB_ phase existence are difficult to establish because mixtures crystalize before the N_TB_ phase appears. The N_TB_-N transition on DSC thermograms is visible only upon second heating. In Figure 8, an example of DSC thermograms for the system CB7CB-9CB are shown. For mixtures containing a 0.5 and 0.6 mole fraction of 9CB, the N_TB_-N transition is not visible because the N_TB_ phase supercools very easily and does not appear under conditions of the DSC measurement. But it was visible during POM measurements; see micrographs of the textures recorded for three mixtures of CB7CB-9CB containing 0.3, 0.4, and 0.5 mole fractions of 9CB given in Figure 9. The change in the enthalpy of the N_TB_-N transition versus concentration shows that it decreases linearly and, for mixtures of 0.5 and 0.6 of 9CB, should be smaller than 0.1 kJ/mol. Mixtures containing CB7CB supercool to low temperatures, the easiest of all mixtures containing CBnCB compounds. This is why it is possible to measure the properties of the monotropic N_TB_ phase in a broad temperature–concentration range.

**Figure 10:**
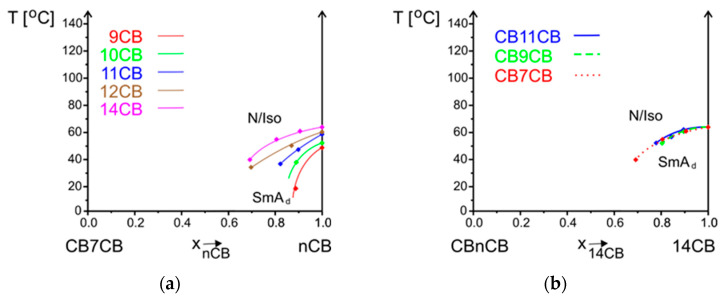
Comparison of lines corresponding to phase equilibrium SmA_d_-N/Iso for systems (**a**) CB7CB-nCB (n = 9–12), 14; (**b**) CBnCB-14CB (n = 7, 9, 11).

Regarding the **SmA_d_ phase** which is formed by nCB with n = 8–12, 14, the maximum temperature of the existence of this phase increases as the alkyl chain length of the cyanobiphenyls increases (Table 1). The same is observed in mixtures with bismesogens (Figure 10a). The range of the concentration of the existence of this phase increases and the maximum temperature is not destabilized so rapidly. However, it is hard to establish how close the N_TB_ phase is to the SmA_d_ phase because the samples crystalize before reaching temperatures in which it may be observed (Figure 5e–j). The phase diagram presented by Aouini et al. [34] for the CB7CB-8CB system was enriched with the results of dielectric measurements. The equilibrium lines N_TB_-N and SmA_d_-N were very close to each other, but the authors could not confirm the transition between the N_TB_ and SmA_d_ phases.

#### 3.2.2. Influence of Alkyl Chain Length of CBnCB on Phase Situation

**Figure 11:**
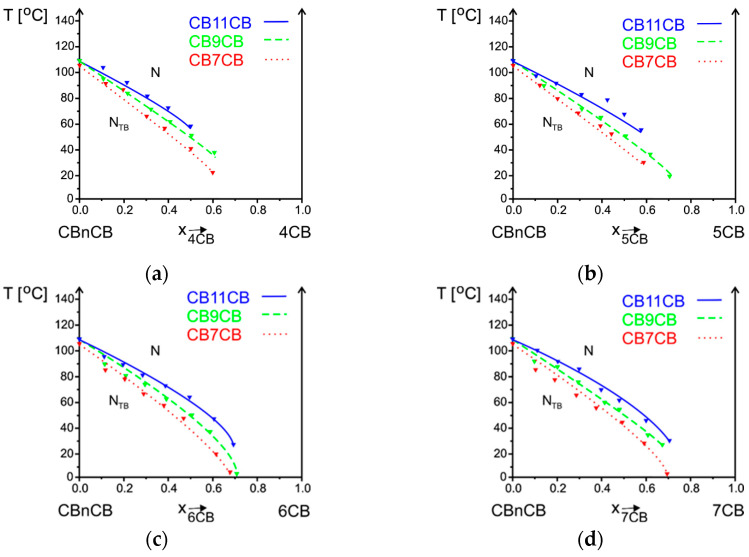

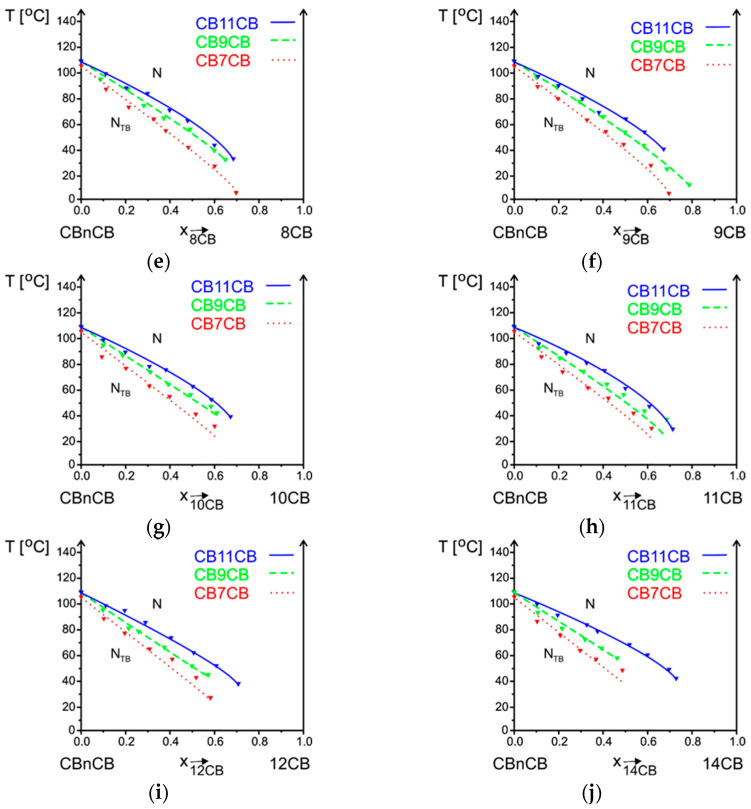
Influence of the length of the alkyl chain of bismesogen CBnCB n = 7, 9, 11 in mixtures with nCB (n = 4 (**a**), 5 (**b**), 6 (**c**), 7 (**d**), 8 (**e**), 9 (**f**), 10 (**g**), 11 (**h**), 12 (**i**), 14 (**j**)) on phase transition N_TB_-N temperatures versus concentration x (mole fraction).

The influence of the alkyl chain length of bismesogens CB7CB, CB9CB, and CB11CB in mixtures with nCB on N_TB_-N transition temperature was determined (Figure 11). In all systems, an increase in the number of methylene groups (n = 7, 9, 11) in the alkyl chain of bismesogen causes an increase in the N_TB_-N transition temperatures. The maximum concentration for which this phase transition is observed is between the 0.5 and 0.8 mole ratio of nCB. We may assume that the concentration limit is set at a satisfactory level for the systems where the line corresponding to N_TB_-N phase equilibrium reached 0 °C. In all other cases, the samples were crystallized before the monotropic phase was reached; thus, this transition was not possible to be measured for a bigger concentration of cyanobiphenyl.

The influence of the linking chain length on the SmA_d_ phase is presented in Figure 10b, where lines corresponding to the phase equilibrium SmA_d_-N/Iso are presented. Their slopes are the same, which may suggest that the mesogenic units build in the smectic layer structure regardless of the spacing length. In particular, bismesogens can form the SmA phase when the linking group is sufficiently long [22].

### 3.3. XRD Studies of Mixtures CB7CB with 9CB and 4CB

The X-ray diffraction results for pure CB7CB compounds are already reported in the literature [14]. The two reflexes 4.5 Å and 12 Å are present on diffractograms. Taking into account that the molecular length of this molecule is 26 Å, the former reflex corresponds to the average distance between molecules and the latter indicates the intercalated structure [22]. The rigid arms of bismesogens are ordered side by side but in such a way that one arm is adjacent to one and the other to another bismesogen.

**Figure 12:**
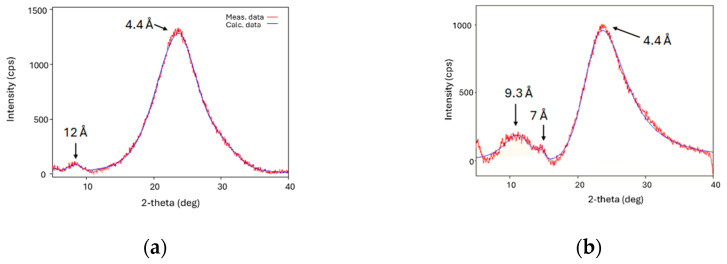
X-ray diffractograms for mixtures: (**a**) CB7CB-9CB; (**b**) CB7CB-4CB. Both mixtures contain 0.5 mole fraction of cyanobiphenyl. Measurement temperature is 23 °C.

We studied two mixtures, CB7CB-9CB and CB7CB-4CB, both of which contained a 0.5 mole fraction of cyanobiphenyl. The results of the studies are presented in Figure 12. For both systems, reflexes corresponding to the average separation of molecules (4.4 Å) are observed. The correlation length calculated from the Scherrer equation is 13.5 Å for this signal, which corresponds to three neighboring molecules. For a mixture containing CB7CB with 9CB, the second reflex is exactly of the same value as for pure bismesogen and is close to half of the length of the CB7CB molecule (12Å). This suggests that molecules with a longer tail adopt the structure of the CB7CB host molecules. The calculated correlation length is 38.2 Å, three times bigger than the measured distance. For a mixture containing the 4CB compound, the small angle reflex has two maxima. The reflex giving a distance of 9.3 Å corresponds to the length of the cyanobiphenyl group of both compounds. The next small reflex giving a distance of 7.0 Å corresponds to the length of the linking group of CB7CB. This may suggest that the presence of 4CB molecules causes the segregation of rigid and flexible parts, even more than in pure bismesogen.

## 4. Conclusions

Ten homologs of cyanobiphenyls nCB (n = 4–12, 14) were added to structurally similar bismesogens CBnCB (n = 7, 9, 11), forming a nematic twist-bend phase. They are good candidates as components for mixtures showing this phase because they support this phase in a broad concentration range (from 0.5 to 0.8 mole fraction). It is worth noticing that the slope of the equilibrium curves N_TB_-N is the same regardless of the length of cyanobiphenyl. Slightly bigger N_TB_ phase stability occurs when the alkyl chain length of cyanobiphenyl is of the same length as the linking alkyl chain of bismesogen CBnCB. On the other side, an increase in the length of the linking alkyl chain of a bismesogen causes an increase in the N_TB_ phase stability both for pure compounds and their mixtures with cyanobiphenyls. The biggest problem with the N_TB_ phase is that this phase is mostly monotropic. Unfortunately, the melting point is not lowered by nCB compounds as much as predicted. Because the rigid parts of bismesogen and monomesogen are the same, molecules in deficiency incorporate into the arrangement of molecules in excess. This is seen both for the nCB built in the structure of the N_TB_ of CBnCB and for the CBnCB built in the structure of SmA_d_ of nCB. The slope of equilibrium curves on the phase diagrams is the same, regardless of the alkyl chain length. When molecules of cyanobiphenyls are short, they cause the segregation of rigid and flexible parts (mixture CB7CB-4CB), in contrast to the CB7CB-9CB mixture, where longer molecules of cyanobiphenyls better fit the structure.

It is expected that the N_TB_ phase will be obtained at room temperature as an enantiotropic phase. New chemical structures can be searched for, but it is much easier to do this by formulating multicomponent mixtures in which the melting point is lowered. Miscibility studies allow for the determination of which compounds are favorable for the formation of such mixtures. Further studies will be conducted on the influence of the chemical structure of monomesogens, e.g., the core structure or the type of terminal groups, on the behavior of the N_TB_ phase in mixtures.

## Figures and Tables

**Table 1:** Acronyms and phase transition temperatures (°C) of the tested compounds measured in this research by POM method. Temperatures in brackets denote monotropic transition. These compounds were first reported in refs. presented in the last column.

Acronym	Cr		N_TB_		SmA_d_		N		Iso	Refs.
CB7CB	*	102.9 ^1^	*	104.4	-	-	*	117.2	*	[14,37]
CB9CB	*	84.9	*	107.8	-	-	*	123.4	*	[38,39]
CB11CB	*	105.7 ^2^	*	108.7	-	-	*	125.4	*	[40]
4CB	*	49.5	-	-	-	-	*	(16.5)	*	[41]
5CB	*	21.6	-	-	-	-	*	39.4	*	[41]
6CB	*	14.8	-	-	-	-	*	29.5	*	[41]
7CB	*	30.5	-	-	-	-	*	43.3	*	[41]
8CB	*	20.3	-	-	*	33.0	*	39.7	*	[41]
9CB	*	43.9	-	-	*	48.4	*	50.3	*	[41]
10CB	*	44.5	-	-	*	51.7	-	-	*	[42]
11CB	*	54.3	-	-	*	58.3	-	-	*	[42]
12CB	*	47.4	-	-	*	59.9	-	-	*	[42]
14CB	*	58.0	-	-	*	63.6	-	-	*	[43]

^1^ from DSC: melting of Cr_3_ 66.9, of Cr_2_ 102.5, of Cr_1_ 105.2; N_TB_ 103.1 N 115.7 Iso. ^2^ from DSC: melting of Cr_2_ 96.0, of Cr_1_ 104.7; N_TB_ 108.2 N 124.9 Iso.

## Data Availability

Data are contained within the article.
